# Laropiprant Attenuates EP3 and TP Prostanoid Receptor-Mediated Thrombus Formation

**DOI:** 10.1371/journal.pone.0040222

**Published:** 2012-08-01

**Authors:** Sonia Philipose, Viktoria Konya, Mirjana Lazarevic, Lisa M. Pasterk, Gunther Marsche, Sasa Frank, Bernhard A. Peskar, Akos Heinemann, Rufina Schuligoi

**Affiliations:** 1 Institute of Experimental and Clinical Pharmacology, Medical University of Graz, Graz, Austria; 2 Institute of Molecular Biology and Biochemistry, Medical University of Graz, Graz, Austria; Heart Center Munich, Germany

## Abstract

The use of the lipid lowering agent niacin is hampered by a frequent flush response which is largely mediated by prostaglandin (PG) D_2_. Therefore, concomitant administration of the D-type prostanoid (DP) receptor antagonist laropiprant has been proposed to be a useful approach in preventing niacin-induced flush. However, antagonizing PGD_2_, which is a potent inhibitor of platelet aggregation, might pose the risk of atherothrombotic events in cardiovascular disease. In fact, we found that *in vitro* treatment of platelets with laropiprant prevented the inhibitory effects of PGD_2_ on platelet function, i.e. platelet aggregation, Ca^2+^ flux, P-selectin expression, activation of glycoprotein IIb/IIIa and thrombus formation. In contrast, laropiprant did not prevent the inhibitory effects of acetylsalicylic acid or niacin on thrombus formation. At higher concentrations, laropiprant by itself attenuated platelet activation induced by thromboxane (TP) and E-type prostanoid (EP)-3 receptor stimulation, as demonstrated in assays of platelet aggregation, Ca^2+^ flux, P-selectin expression, and activation of glycoprotein IIb/IIIa. Inhibition of platelet function exerted by EP4 or I-type prostanoid (IP) receptors was not affected by laropiprant. These *in vitro* data suggest that niacin/laropiprant for the treatment of dyslipidemias might have a beneficial profile with respect to platelet function and thrombotic events in vascular disease.

## Introduction

Prostanoids are important regulators of platelet function and are involved in hemostasis by differentially influencing platelet aggregation [1]. The endothelium and, under inflammatory conditions, infiltrating leukocytes, vascular smooth muscle cells, and activated platelets release prostaglandins (PG) such as thromboxane (TX) A_2_, PGI_2_, PGE_2_ and PGD_2_ [2]–[7]. Platelets express corresponding receptors for these prostaglandins [1].

While TXA_2_ by activating the TP receptor is a very potent inducer and amplifier of platelet aggregation, PGI_2_ and PGD_2_ are clearly anti-aggregatory in their action. In contrast, PGE_2_ evokes a biphasic response, at nanomolar concentrations facilitating, while at micromolar concentrations inhibiting platelet aggregation [8]–[11]. The pro-aggregatory effect of PGE_2_ has been ascribed to the activation of the E-type prostanoid (EP) 3 receptor [12], [13]. An EP3 antagonist has been proposed to be useful for antithrombotic therapy [12]. Our group and others have simultaneously shown that the anti-aggregatory action of PGE_2_ in human platelets is mediated by EP4 receptors and a selective EP4 agonist potently inhibits platelet aggregation, Ca^2+^ mobilization, upregulation of P-selectin, and the activation of glycoprotein (GP) IIb/IIIa [14]–[17]. We could demonstrate that these inhibitory effects of EP4 receptors on platelet activation translate to potent antithrombotic activity as shown by an *in vitro* thrombus formation assay using whole blood [14].

Niacin has been shown to improve all lipoprotein abnormalities, by lowering cholesterol, triglycerides, low density lipoproteins (LDL), and apolipoprotein(a), while increasing high density lipoproteins (HDL) [18], alone or in combination with statins [19]. However, a frequent adverse effect in patients receiving niacin (1–2 g/day) is the development of significant cutaneous warmth and facial vasodilation. Although flushing is transient following intake of niacin, 5–6% percent of patients discontinue niacin because of that side effect [20]. Recent studies have elucidated the molecular mechanism that mediates niacin-induced flushing: Niacin acting through the G protein-coupled receptor GPR109A stimulates the production of several prostaglandins, including PGE_2_ and PGD_2_, in mast cells, keratinocytes and monocytes/macrophages [21], [22]. Particularly, PGD_2_ acting through the DP receptor has been alleged to cause the niacin-induced flush [23]. Consequently, a combination of the DP receptor antagonist laropiprant with niacin (Tredaptive®) is currently marketed for treatment of dyslipidemias for Europe [24]. In contrast, the U.S. Federal Drug Administration rejected the drug in 2008; although the reasons for the decision have not been published safety concerns are likely to have played a role. Although niacin/laropiprant has been reported to be effective and well tolerated [25]–[28], its effect on thrombotic cardiovascular events, such as myocardial infarction and stroke has not been revealed yet. Since prostaglandins are important regulators of platelet function, laropiprant, by interfering with the anti-aggregatory action of PGD_2_ [10], might confer additional cardiovascular risks to patients, thus outweighing the beneficial effects of niacin on lipid metabolism. However, laropiprant has also been purported to block the thromboxane receptor (TP) at high concentration, but the therapeutic relevance of this finding has not been followed up yet [25].

Prompted by these open issues we investigated the effects of laropiprant and niacin on *in vitro* thrombus formation in flowing human whole blood and found that both compounds have anti-platelet properties. While the inhibitory effect of niacin does not involve prostanoids such as PGD_2_, laropiprant at higher concentrations inhibits platelet function by blocking TP- and EP3-mediated platelet activation. Our results suggest that niacin/laropiprant might have beneficial effects on platelet function.

**Figure 1:**
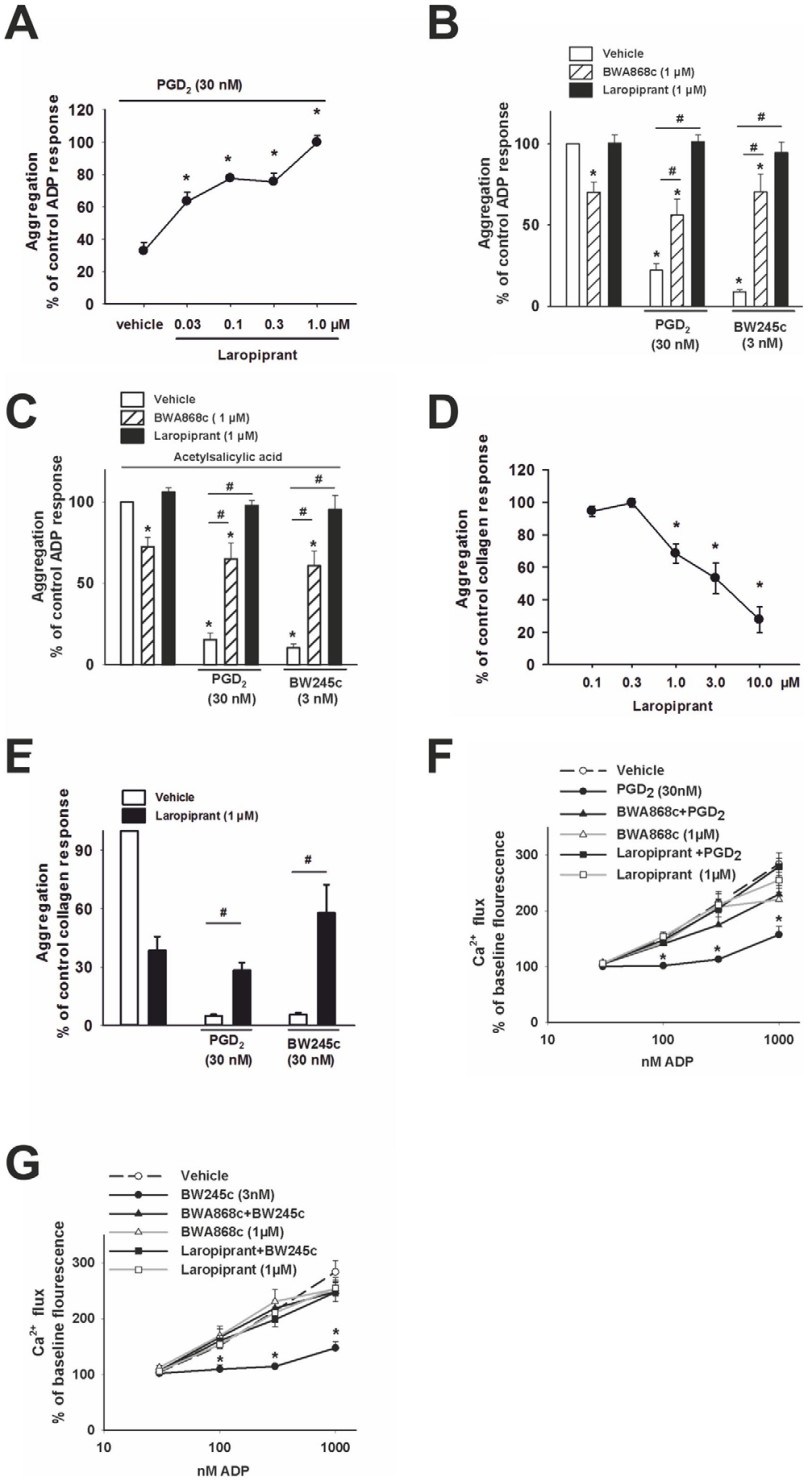
DP receptor activation inhibits platelet aggregation and intracellular Ca^2+^ mobilization, effects that are counteracted by laropiprant. (**A–E**) Aggregation was induced using ADP or collagen at concentrations which were adjusted to give submaximal aggregation. Data were expressed as percent of the control response. (**A**) ADP-induced aggregation was inhibited by PGD_2_ (30 nmol/L) and this effect was concentration dependently counteracted by laropiprant (n = 6). (**B**) The ADP- induced aggregation was slightly reduced by the DP receptor antagonist BWA868c (1 μmol/L), but not by laropiprant (1 μmol/L) alone. PGD_2_ (30 nmol/L) and the DP agonist, BW245c (3 nmol/L) caused pronounced inhibition of platelet aggregation and these effects were reversed by BWA868c and laropiprant (n = 4). (**C**) Pre-treatment of platelets with acetylsalicylic acid (1 mmol/L) did not affect the inhibitory effects of PGD_2_ (30 nmol/L) and BW245c (3 nmol/L) and the reversal of these effects by BWA868c and laropiprant at 1 μmol/L (n = 4). (**D**) Laropiprant caused a concentration-dependent inhibition of collagen-induced aggregation (n = 6). (**E**) Laropiprant (1 μmol/L) counteracted the inhibition of aggregation by PGD_2_ (30 nmol/L) and BW245c (3 nmol/L) (n = 4–6). (**F, G**) Ca^2+^ responses were detected by ﬂow cytometry as changes in ﬂuorescence of the Ca^2+^-sensitive dye Fluo-3 by and are presented as percent of baseline ﬂuorescence. Ca^2+^ flux induced by ADP (30–1000 nmol/L) was significantly reduced by pre-treatment of platelets with (**E**) PGD_2_ (30 nmol/L) and (**F**) BW245c (3 nmol/L). The inhibition of the Ca^2+^ flux by DP receptor activation was reversed by laropiprant (1 μmol/L) and the DP antagonist, BWA868c (1 μmol/L) (n = 6). Values are shown as mean+SEM. *P<0.05 as compared to vehicle and # P<0.05 as compared to agonist treatment.

**Figure 2:**
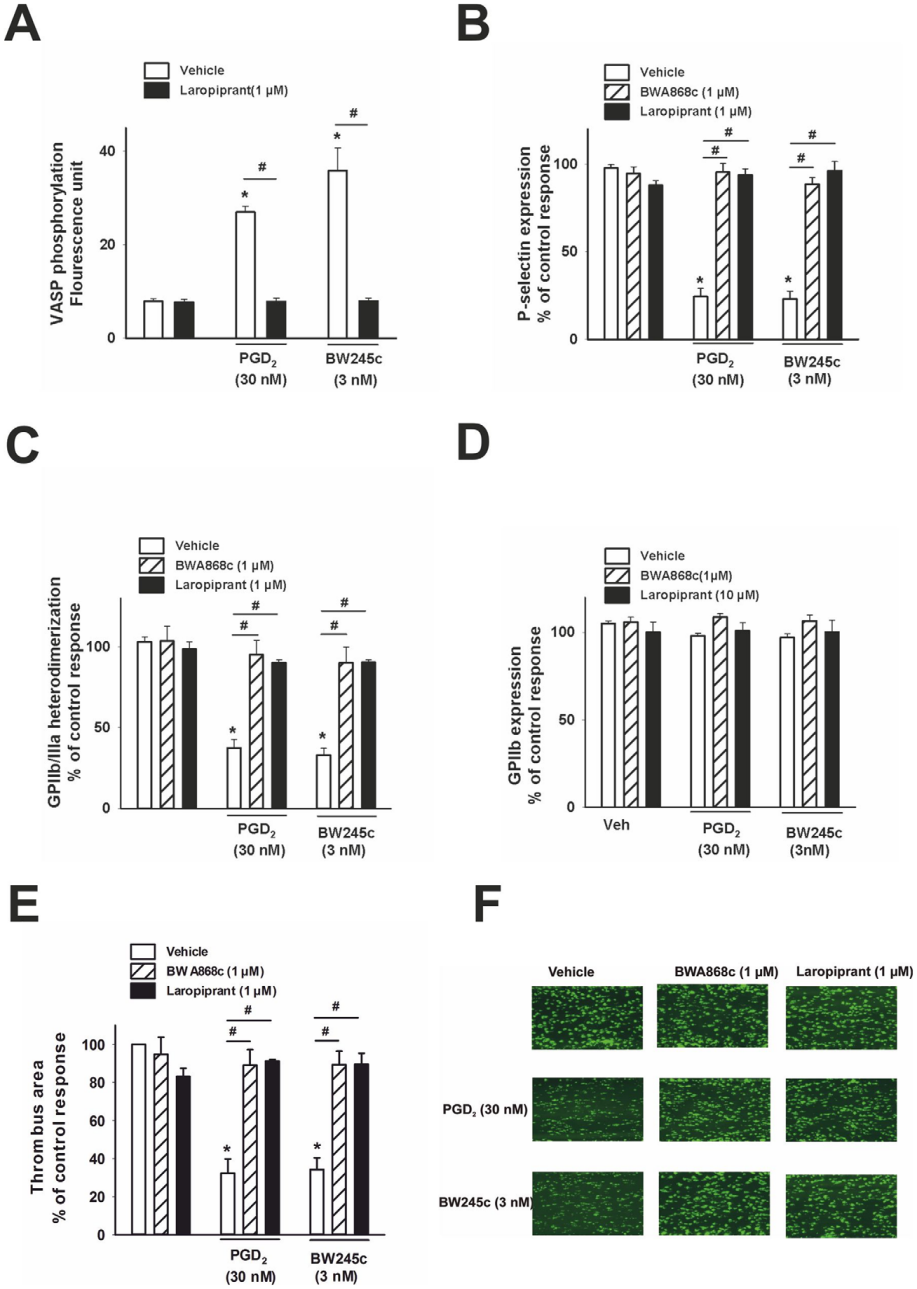
DP receptor activation increases VASP phosphorylation, and inhibits P-selectin expression, GPIIb/IIIa activation and *in vitro* thrombus formation. (A) VASP phosphorylation was visualized using an anti-VASP p-Ser 157 antibody in flow cytometry and data were expressed in fluorescence units. VASP phosphorylation in platelets stimulated with ADP (3 µmol/L) was significantly enhanced by PGD_2_ (30 nmol/L) as well as the DP agonist BW245c (3 nmol/L), which was completely prevented by pre-treatment of platelets with laropiprant (1 μmol/L) (n = 4). (**B**) ADP (3 μmol/L) increased the surface expression of P-selectin, detected using a CD62P antibody, in platelets primed with cytochalasin B (5 μg/mL). PGD_2_ (30 nmol/L) and BW245c (3 nmol/L) caused a significant inhibition of P-selectin expression on the platelet surface and this effect was revoked by pre-treatment with DP antagonists, BWA868c (1 μmol/L) and laropiprant (1 μmol/L; n = 6). (**C**) The ADP (3 μmol/L) induced activation of GPIIb/IIIa, detected using a conformation dependent antibody PAC-1, was attenuated by PGD_2_ (30 nmol/L) and the DP agonist BW245c (3 nmol/L). These effects were reversed by BWA868c and laropiprant at 1 μmol/L (n = 6). (**D**) GPIIb expression was determined using an anti-CD41 antibody by flow cytometry. None of the treatments affected the GPIIb expression. Data were expressed as percent of control ADP response. (**E**) Whole blood incubated with the ﬂuorescent dye 3,3′-dihexyloxacarbocyanine iodide was perfused over collagen-coated channels and thrombus formation was recorded by fluorescence microscopy. Thrombus-covered area was calculated by computerized image analysis and is expressed as percent of control (vehicle) response. Vehicle-treated samples showed pronounced thrombogenesis over collagen. Both PGD_2_ (30 nmol/L) and BW245c (3 nmol/L) markedly decreased the formation of thrombi. The DP antagonist BWA868c and laropiprant (1 µmol/L each) had no effect on thrombus formation by themselves but reversed the inhibition of thrombogenesis by DP receptor stimulation (n = 4). (F) Original fluorescence images. Values are shown as mean+SEM. *P<0.05 as compared to vehicle and # P<0.05 as compared to agonist treatment.

**Figure 3:**
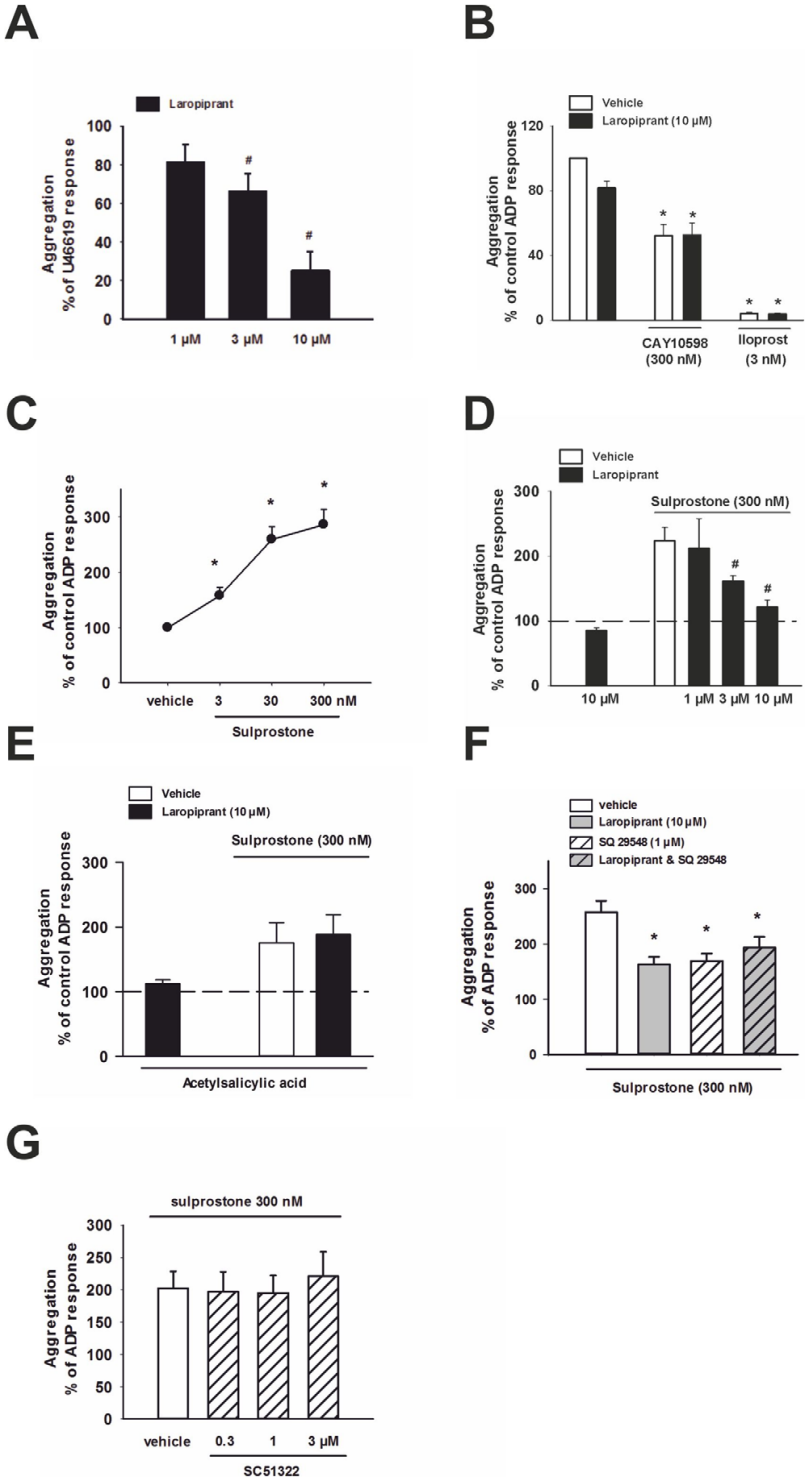
Laropiprant antagonizes the increased platelet aggregation by TP and EP3 receptor activation In (**A**), aggregation was induced by U46619 (300 nmol/L) which was concentration-dependently inhibited by laropiprant (n = 4). In **B–G**, ADP concentrations (1.25–10 μmol/L) were adjusted to give 30–50% of maximal aggregation. (**B**) The inhibiton of ADP-induced aggregation by the EP4 agonist CAY10598 (300 nmol/L) and the IP agonist iloprost (3 nmol/L) was not affected by laropiprant (10 nmol/L, (n = 7)). (**C**) The EP3 agonist sulprostone concentration dependently amplified ADP-induced aggregation (n = 4–6). (**D**) The effect of sulprostone (300 nmol/L) was concentration dependently inhibited by laropiprant (n = 4). (**E**) Pretreatment with acetylsalicylic acid (1 mmol/L) markedly attenuated the pro-aggregatory effect of sulprostone, and in this case, laropiprant was unable to reverse the stimulatory effect of the EP3 agonist. Data were expressed as percent of control response. (**F**) The TP receptor antagonist SQ29578 (1 µmol/L) inhibited the sulprostone-induced increase in platelet aggregation to the same extend as laropiprant (10 µmol/L). The combination of SQ29578 and laropiprant did not cause further inhibition as compared to laropiprant or SQ29578 alone (n = 4–6) (**G**) The pro-aggregatory effect of sulprostone was not inhibited by the EP1 receptor antagonist SC51322 (n = 4). Data were expressed as percent of control ADP response and are shown as mean+SEM. *P<0.05 as compared to vehicle and^ #^P<0.05 as compared to the respective agonist treatment.

**Figure 4:**
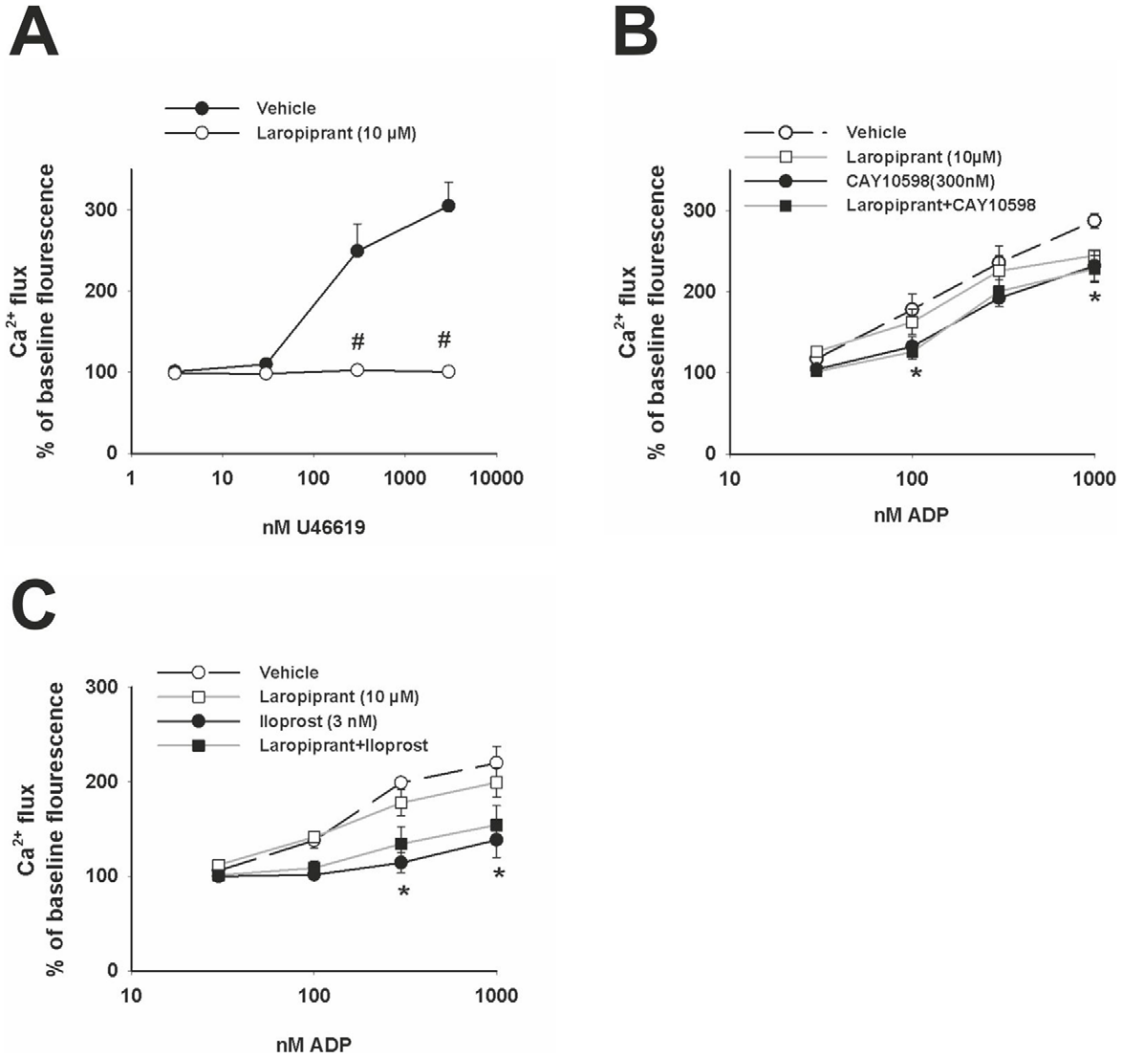
Laropiprant antagonizes Ca^2+^ mobilization induced by TP receptor activation. Ca^2+^ responses were detected by ﬂow cytometry as changes in ﬂuorescence of the Ca^2+^ -sensitive dye Fluo-3 by ﬂow cytometry and are presented as percent of baseline ﬂuorescence. (**A**) The TP agonist, U46619 (3–3000 nmol/L), induced Ca^2+^flux in a concentration-dependent manner and this effect was completely inhibited by laropiprant at 10 μmol/L (n = 6). (**B**) The EP4 receptor agonist CAY10598 (300 nmol/L) and (**C**) the IP receptor agonist iloprost (3 nmol/L) caused a significant inhibition of the ADP-induced Ca^2+^ flux (n = 4). Laropiprant (10 µmol/L) did not antagonize these effects (n = 4). Values are shown as mean+SEM. *P<0.05 as compared to vehicle and ^#^P<0.05 as compared to the respective agonist treatment treatment.

**Figure 5:**
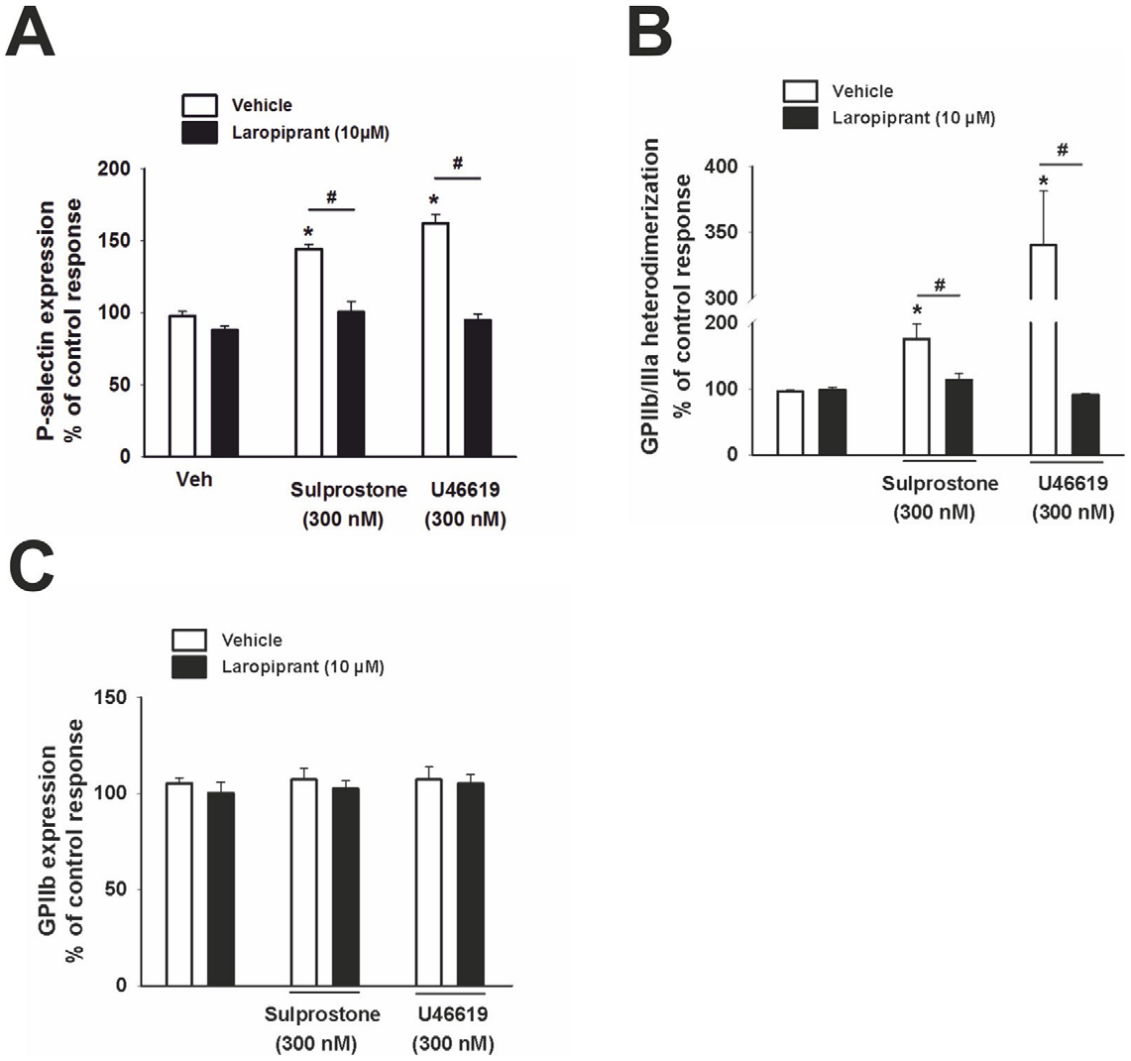
Laropiprant antagonizes the P-selectin expression and GPIIb/IIIa activation induced by TP and EP3 receptor activation. (A) ADP (3 μmol/L) increased the surface expression of P-selectin in platelets primed with cytochalasin B (5 μg/mL), detected using a CD62P antibody. The EP3 agonist sulprostone (300 nmol/L) and U46619 (300 nmol/L) elevated P-selectin expression on the surface of platelets. These effects were counteracted by laropiprant at 10 μmol/L (n = 5). (**B**) The ADP (3 μmol/L) induced activation of GPIIb/IIIa, detected using a conformation-dependent antibody, PAC-1, was increased by sulprostone (300 nmol/L) and U46619 (300 nmol/L). Laropiprant at 10 µmol/L antagonized these effects (n = 5). (**C**) GPIIb expression was determined using an anti-CD41 antibody by flow cytometry. None of the treatments affected the GPIIb expression (n = 5). Data were expressed as percentage of ADP control response and are shown as mean+SEM. *P<0.05 as compared to vehicle and # P<0.05 as compared to the respective agonist treatment.

**Figure 6:**
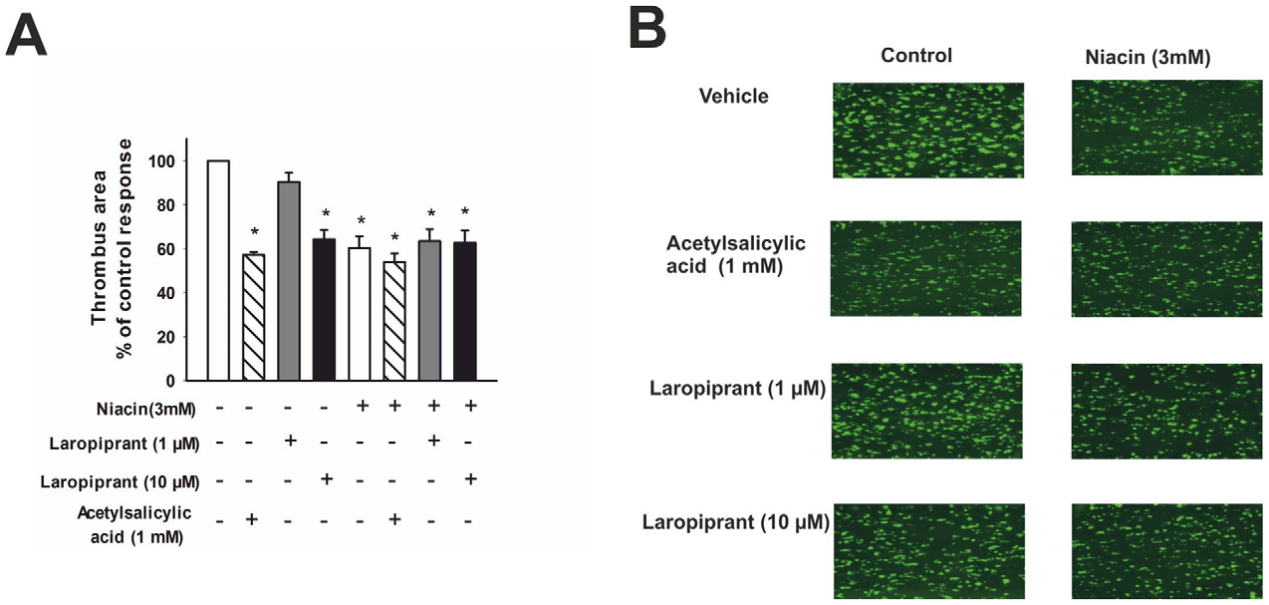
Laropiprant at 10 µmol/L and niacin inhibit *in vitro* thrombus formation. Whole blood was incubated with the ﬂuorescent dye 3,3′-dihexyloxacarbocyanine iodide, perfused over collagen-coated channels, and thrombus formation was recorded by ﬂuorescence microscopy. The images were taken 3 minutes after the start of the perfusion and are representative of 4 different experiments. (**A**) Vehicle-treated samples showed pronounced and rapid thrombogenesis over collagen. Treatment of whole blood with acetylsalicylic acid (1 mmol/L), laropiprant 10 µmol/L and niacin 3 mmol/L for 30 min, caused a marked reduction of thrombus formation, while laropiprant 1 µmol/L had no effect. The antithrombotic effect of niacin was not influenced by pretreatment of blood with acetylsalicylic acid or laropiprant. Thrombus-covered area was calculated by computerized image analysis and is expressed as percent of control (vehicle) response. (**B**) Original fluorescence images. Values are shown as mean+SEM. *P<0.05 versus vehicle.

## Methods

### Ethics statement

The study was approved by the Institutional Review Board (Ethics committee of the Medical University Graz). Blood was drawn from healthy volunteers after they signed an informed consent form.

### Material

All laboratory reagents were from Sigma (Vienna, Austria), unless specified. Assay buffer as used in Ca^2+^ flux and flow cytometric immunostaining was Dulbecco's modified phosphate-buffered saline (PBS; with or without 0.9 mM Ca^2+^ and 0.5 mM Mg^2+^; Invitrogen, Vienna, Austria). Laropiprant, sulprostone, PGD_2_, BW245c, BWA868c, U46619, CAY10598 and iloprost were purchased from Cayman (Ann Arbor, MI, USA). Fixative solution was prepared by adding 9 ml distilled water and 30 mL FACS-Flow to 1 mL CellFix. ADP and equine fibrillar collagen were obtained from Probe&Go (Osburg, Germany). Agonists/antagonists were dissolved in water, ethanol or dimethyl sulfoxide (DMSO) and further diluted in assay buffer to give a final concentration of the solvents <0.1%. CD62P-FITC and PAC-1-FITC antibodies were obtained from Becton Dickenson (Vienna, Austria), CD41 antibody from Invitrogen and VASP (pSer 157)-FITC from Acris Antibodies (Herford, Germany). D, L-lysin acetylsalicylic acid was obtained from Bayer (Munich, Germany).

### Platelet aggregation

Platelet-rich and platelet-poor plasma were prepared from citrated whole blood by centrifugation and aggregation was performed by addition of ADP, collagen or U46619 in an Aggrecorder II (KDK Corp, Kyoto, Japan) [14], [29]–[31]. CaCl_2_ at a final concentration of 1 mmol/L was added 2 min before ADP or collagen [32]. In some experiments, acetylsalicylic acid (1 mmol/L as lysine acetylsalicylic acid) was added to platelet-rich plasma 30 minutes before the proaggregatory stimulus. To measure inhibition or enhancement of aggregation, the agonists were added 5 min before the Ca^2+^ stimulus. Antagonists were added 10 min before the agonists. Data were expressed as percentage of maximum light transmission, with nonstimulated platelet-rich plasma being 0% and platelet-poor plasma 100%. The used concentrations of laropiprant were within the peak concentrations obtained by oral administration of the clinically used dose (40 mg/day) or within the peak concentration of a well-tolerated high dose (200 mg/day) in patients [25].

### Ca^2+^ flux

Intracellular Ca^2+^ levels were analyzed by flow cytometry [33]. Platelet rich plasma was loaded with 5 µmol/L of the acetoxymethyl ester of Fluo-3 in the presence of 2.5 mmol/L probenecid at 37°C for 30 minutes and were then resuspended in Tyrode's buffer (134 mM NaCl, 1 mM CaCl_2_, 12 mM NaHCO_3_, 2.9 mM KCl, 0.34 mM Na_2_HPO_4_, 1 mM MgCl_2_ and 0.055 mM glucose, supplemented with 10 mM HEPES). Changes in intracellular free Ca^2+^ levels in response to ADP (30 to 1000 nmol/L) were detected as increase in fluorescence intensity of the Ca^2+^-sensitive dye Fluo-3 in the FL-1 channel. Data were expressed as percent change of baseline.

### Detection of vasodilator stimulatory phosphoprotein (VASP) phosphorylation

Platelet rich plasma was suspended in PBS containing Ca^2+^ and Mg^2+^. Platelets were pre-treated with the antagonist/vehicle for 5 min followed by agonist/vehicle treatment for 5 min before stimulation with 3 μM ADP for another 5 min. The samples were fixed by adding twice the volume of 2% formaldehyde for 10 min. PBS without Ca^2+^ and Mg^2+^ was added to the reaction mixture and then centrifuged at 400 g for 7 min. Thereafter, platelets were permeabilized by adding 0.5% triton X-100 and incubated for 10 min washed with PBS without Ca^2+^ and Mg^2+^ and stained using the VASP pSer 137 FITC-conjugated antibody (1 μg/ml in antibody diluent) for 30 min at room temperature in the dark [34]. After washing and fixation (with fixation solution as described in materials) the samples were analyzed by flow cytometry. Data were expressed in fluorescence units.

### Flow cytometric immunofluorescence staining of P-selectin and GPIIb/IIIa

Platelet rich plasma was suspended in PBS containing Ca^2+^ and Mg^2+^ for immunofluorescence staining. Incubation of the platelets with prostanoids/agonists was done for 5 min. Pretreatment with antagonists started 10 min, and with acetylsalicylic acid 30 min, before the prostanoid/agonist treatment. For P-selectin staining platelets were activated by ADP (3 μM) and cytochalasin B (5 μg/ml) for 15 min at 37°C in the presence of the anti-CD62P-FITC onjugated antibody [14], [35]. The samples were washed and fixed, and P-selectin upregulation was detected by flow cytometry.

Activation of the fibrinogen receptor GPIIb/IIIa was assessed using the PAC-1-FITC conjugated antibody that recognizes a conformation-dependent determinant on the GPIIb/IIIa complex [36]. Total receptor expression was determined with an anti-CD41 FITC-conjugated antibody directed against GPIIb alone. For staining after the pre-treatment with antagonist/agonist, stimulation with ADP (3 μM) was carried out at 37°C for 5 min in the presence of the antibody. Samples were washed and fixed, and then analyzed by flow cytometry [14], [36]. Data were expressed as percent of control (vehicle) response.

### *In Vitro* thrombogenesis

Vena8Fluoro+ Biochips (Cellix, Dublin, Ireland) were coated with collagen (200 µg/mL) at 4°C overnight and thereafter blocked with bovine serum albumin (10 µg/mL) for 30 minutes at room temperature followed by washing steps. Whole blood collected in sodium citrate was incubated with 3, 3-dihexyloxacarbocyanine iodide (1 µmol/L) in the dark for 10 minutes [14]. PGD_2_ (30 nmol/L), BW245c (3 nmol/L) were added 10 min before the start of perfusion, and the DP antagonist BWA868c or laropiprant (1 µmol/L) were added 10 min before the agonists. In another set of experiments whole blood was treated with niacin (3 mmol/L), acetylsalicylic acid (1 mmol/L) or laropiprant (1 µmol/L and 10 µmol/L) for 30 min. CaCl_2_ at a final concentration of 1 mmol/L was added 2 minutes before the perfusion over the collagen-coated chip. Perfusion was carried out at a shear rate of 30 dynes cm^2^. Thrombus formation was recorded by a Zeiss Axiovert 40 CFL microscope, Zeiss A-Plan 10X/0.25 Ph1 lens using Hamamatsu ORCA-03G digital camera and Cellix VenaFlux software. Computerized image analysis was performed by DucoCell analysis software (Cellix, Dublin), where the area covered by the thrombus was calculated. Data were expressed as percent of area covered in a control sample.

### Statistical analysis

Data are shown as mean+SEM for n observations. Repeated measurements were performed using blood from the same healthy individual. Comparisons of groups were performed using Wilcoxon signed rank test or one-way ANOVA for repeated measurements with two sided – Dunnett's post test or Bonferroni's test. Probability values of P<0.05 were considered as statistically significant.

## Results

### Laropiprant, an antagonist for DP receptor-mediated inhibition of platelet function

PGD_2_ is known to inhibit platelet aggregation via activation of the DP receptor. First we investigated whether laropiprant counteracted the effects of PGD_2_ and the DP agonist BW245c, on platelet aggregation. Platelet rich plasma was treated with PGD_2_ (30 nmol/L, a concentration which corresponds with the EC50 of the prostanoid [30]) or the DP agonist BW245c (3 nmol/L [37]) for 7 min. Platelet aggregation was induced with ADP (1.5–10 µmol/L) to give a submaximal effect (50– 60% aggregation). PGD_2_ (30 nmol/L) caused a significant inhibition of aggregation that was concentration dependently counteracted by laropiprant (with a maximal inhibition seen at 1 µmol/L Figure 1 A, B) and the selective DP antagonist BWA868c (1 µmol/L, Figure 1 B). Also the significant inhibition of aggregation caused by BW245, was abrogated by BWA868c (1 µmol/L) as well as by laropiprant (1 µmol/L; Figure 1 B). While laropiprant on its own showed no effect, BWA868c caused a slight inhibition of ADP- induced aggregation (Figure 1 B) which is consistent with the known intrinsic activity of BWA868c. To exclude the possibility that the inhibitory effect of PGD_2_ or BW245c was due to modulation of prostanoid release from platelets, samples were pretreated with acetylsalicylic acid (1 mmol/L). Acetylsalicylic acid treatment caused a slight reduction of the ADP-induced aggregation (−20.3±7.9%, as compared to non- treated samples; n = 4), but the effects of PGD_2_, BW245c, as well as the inhibition by BW868c or laropiprant were unaffected (Figure 1 C). When platelet aggregation was induced by collagen at a concentration that resulted in a 50–60% aggregation (1.25 to 10 mg/L), we found that laropiprant already at a concentration of 1 µmol/L caused a significant inhibition of the aggregation (Figure 1 D, E) but still counteracted the pronounced inhibition caused by PGD_2_ (30 nmol/L) and BW245c (3 nmol/L) (Figure 1 E).

Elevation of intracellular free Ca^2+^ is an important prerequisite for platelet activation subsequently leading to cytoskeletal changes and subserving platelet aggregation. We investigated whether laropiprant affects the DP receptor-mediated inhibition of Ca^2+^ responses using a flow cytometric Ca^2+^ flux assay. ADP caused a concentration dependent increase in Ca^2+^ flux that was inhibited by PGD_2_ (30 nmol/L) as well as by the DP agonist BW245c (3 nmol/L). These effects were completely reversed by pretreatment of platelets with the DP antagonist BWA868c as well as by laropiprant (each 1 µmol/L) (Figure 1 F, G).

### Laropiprant blocks DP receptor-dependent increase in VASP phosphorylation, as well as inhibition of P-selectin expression, GPIIb/IIIa activation and *in vitro* thrombus formation

VASP is a key regulator for rapid dynamic changes in the cytoskeleton of platelets [38] and is phosphorylated on Ser157 in a cAMP-dependent manner. This phosphorylation leads to conformational changes of cell surface molecules and hence constitutes an important inhibitory pathway of platelet aggregation. It is known that DP receptor activation leads to Gs-mediated increases in intracellular cAMP [39]. Therefore, the effect of DP receptor activation on VASP phosphorylation was investigated. PGD_2_ (30 nM) or BW245c (3 nM) significantly enhanced VASP phosphorylation in platelets stimulated with ADP (3 µmol/L). The DP antagonists BWA868c (n = 4; data not shown) and laropiprant (1 µmol/L each) had no effect alone, but completely reversed the effect of DP receptor stimulation (Figure 2 A).

Since elevation of intracellular free Ca^2+^ is essential for the upregulation of P-selectin and activation of GPIIb/IIIa, we further investigated the effects of DP receptor activation on these responses. Platelets were treated with ADP (3 µmol/L) and cytochalasin (5 µg/mL) to stimulate P-selectin expression on the surface of platelets as determined by an anti-CD62P antibody. The ADP-induced activation of GPIIb/IIIa was detected by staining with the conformation-dependent antibody PAC-1. Both PGD_2_ (30 nmol/L) and BW245c (3 nmol/L) effectively prevented P-selectin expression as well as GPIIb/IIIa activation (Figure 2 B, C). The DP antagonist BWA868c and laropiprant alone (each 1 µmol/L) were without an effect but completely prevented the effect of DP receptor stimulation. The total amount GPIIb/IIIa was not changed by any of these treatments as determined using an anti-CD41 antibody (Figure 2 D).

Platelet adhesion is the first step in thrombus formation and this is governed by (i) platelet-endothelial-leukocyte interactions mediated by P-selectin and (ii) by platelet-platelet interactions mediated by activated GPIIb/IIIa which act as fibrin receptors. Since PGD_2_ via DP receptor activation inhibited both processes, we next assessed whether DP receptor activation also prevented thrombogenesis *in vitro*. Whole blood was perfused through collagen-coated channels at a shear rate of 30 dynes/cm^2^, and thrombus formation was recorded by fluorescence microscopy. PGD_2_ (30 nmol/L) as well as BW245c (3 nmol/L) effectively inhibited thrombogenesis. This inhibition was counteracted by BWA868c and by laropiprant (both 1 µmol/L), at concentrations at which none of them showed significant effects on thrombus formation by themselves (Figure 2 E, F).

These data showed that activation of the DP receptor attenuates platelet aggregation by inhibiting Ca^2+^ mobilization, increasing VASP phosphorylation and inhibition of P-selectin expression and GPIIb/IIIa activation, translating to inhibition of thrombogenesis. These effects were prevented by laropiprant as a DP receptor antagonist.

### Laropiprant prevents TP and EP3 receptor-mediated platelet responses

In addition to its antagonistic effect on the DP receptor, laropiprant purportedly binds to the TP receptor, albeit with a lower affinity [25]. However, it is not known whether laropiprant acts also on other prostanoid receptors. First we investigated the effect of laropiprant on platelet aggregation induced by the TP agonist, U46619. At 300 nmol/L, U46619 caused a pronounced increase in aggregation, which was concentration-dependently inhibited by laropiprant (Figure 3 A). In further experiments, we addressed the possibility that laropiprant in addition to its effect on DP and TP receptors also affects EP3, EP4 and IP receptor-mediated responses. The EP4 agonist CAY10598 (300 nmol/L) significantly reduced, and the EP1/IP agonist iloprost (3 nmol/L) almost completely prevented, the ADP-induced platelet aggregation. These effects were not influenced by laropiprant up to 10 µmol/L, (Figure 3 B). The EP3 agonist sulprostone caused a concentration-dependent increase of ADP-induced platelet aggregation (Figure 3 C) and the response to 300 nmol/L of sulprostone was concentration-dependently inhibited by laropiprant (Figure 3 D). Pretreatment of platelets with acetylsalicylic acid diminished the proaggregatory effect of sulprostone by 70%, and rendered laropiprant ineffective in preventing the remaining sulprostone response (Figure 3 E). Additional experiments showed that the enhanced aggregation induced by sulprostone was also inhibited by the TP receptor antagonist SQ29548 (1 µmol/L) and the combination of laropiprant and SQ29548 caused no further attenuation (Figure 3 F). Since sulprostone can also activate the EP1 receptor in addition to its effect on the EP3 receptor, we employed the EP1 antagonist SC51322 which, however, showed no inhibitory effect towards sulprostone (Figure 3 G). To further elucidate the role of laropiprant we investigated the TP receptor-induced Ca^2+^ flux. U46619 caused a concentration-dependent increase in Ca^2+^ flux which was prevented by pretreating platelets with laropiprant at 10 µmol/L (Figure 4 A). In contrast, laropiprant at 10 µmol/L did not reverse the inhibition of ADP-induced Ca^2+^ flux by the EP4 agonist CAY10598 (300 nmol/L) or the IP agonist iloprost (3 nmol/L) (Figure 4 B, C).

Activation of the EP3 receptor by sulprostone (300 nmol/L) and of the TP receptor by U46619 (300 nmol/L) enhanced the ADP- induced P-selectin surface expression and GPIIb/IIIa activation. Importantly, both effects were totally prevented by laropiprant (10 µmol/L; Figure 5 A, B). The total amount of GPIIb was not changed by any of these treatments as determined using an anti-CD41 antibody (Figure 5 C). Thus in total, laropiprant reversed TP- and EP3-mediated platelet responses, such as platelet aggregation, P-selectin expression, GPIIb/IIIa activation and Ca^2+^ flux.

### Effect of laropiprant and niacin on *in vitro* thrombus formation

Based on the results that laropiprant inhibited the DP, TP and EP3 mediated effects, we investigated how this translates to thrombus formation in whole blood. While laropiprant at 1 µmol/L had no effect on thrombus formation, it caused a pronounced inhibition at 10 µmol/L, comparable to the inhibition of thrombogenesis by acetylsalicylic acid (1 mmol/L) (Figure 6). It has been shown that niacin and its metabolite niceritrol inhibit platelet aggregation *in vitro* [40], [41] and cause the release of TXA_2_, PGD_2_ and PGE_2_ at a clinically relevant concentration of 3 mmol/L [41]. Therefore, we investigated the effect of niacin on thrombus formation. Incubation of whole blood with niacin (3 mmol/L for 30 min) caused a pronounced inhibition of thrombus formation, an effect that was not influenced by pretreatment of blood with acetylsalicylic acid (1 mmol/L) or laropiprant (10 µmol/L). This suggests that – in contrast to the flushing response – formation of prostaglandins, e.g. PGD_2_, are not involved in the inhibitory effect of niacin on platelet aggregation.

## Discussion

In this study we investigated the effects of niacin and laropiprant, the two active substances in the lipid-lowering drug Tredaptive®, on platelet function *in vitro,* in order to address concerns that the drug may harbor the risk of atherothrombotic events. In addition to mediating the “flush” response to niacin, PGD_2_ is a potent anti-aggregatory agent in platelets via activation of the DP receptor [42]. Lipocalin-type PGD_2_ synthase, one of the key enzymes responsible for the biosynthesis of PGD_2_ has been shown to be expressed in vascular endothelial cells [43] in serum of atherosclerotic patients and to be accumulated in the atherosclerotic plaque of coronary arteries with severe stenosis [44], [45]. Therefore, it can be assumed that PGD_2_ is formed in atherosclerotic lesions and may prevent the adhesion and subsequent aggregation of platelets. Moreover, studies have shown that niacin intake increases the plasma concentrations of PGD_2_, PGI_2_ and TXA_2_ [46]–[49] and that the increase in PGD_2_ is by far the most pronounced. Accordingly, laropiprant might prevent the anti-thrombotic effect of PGD_2_ thereby fostering vascular events such as stroke or myocardial infarction.

PGD_2_, apart from being released from mast cells [50], [51], dendritic cells, eosinophils, and Th2 cells [52]–[55], is also synthesized in the vasculature, i.e. by endothelial cells, macrophages and platelets [6], [56]–[59]. PGD_2_ exerts it biological effects via two G protein-coupled receptors, the DP receptor and chemoattractant receptor homologous molecule expressed on Th2 cells (CRTH2). Activation of the DP receptor [30], [60] causes an increase in cAMP production [61] and inhibits Ca^2+^ mobilization in platelets [62]. In this study we further elucidated the cellular mechanisms involved in PGD_2_ inhibition of platelet aggregation and confirmed the potent antagonist effect of laropiprant on the DP receptor. We found that activation of the DP receptor inhibited ADP- and collagen induced platelet aggregation, without involving other inhibitory prostanoid receptors or modulating TXA2 release, since identical results were obtained in acetylsalicylic acid-treated platelets. Moreover, the ADP-induced increase in Ca^2+^ mobilization was abrogated by DP receptor activation, which is in line with previously published results [62]. VASP phosphorylation, which is an indicator of inhibition of platelet aggregation by preventing shape change in platelets [63], [64], was increased by DP receptor activation. Since VASP phosphorylation was assessed at Ser 157, this can be attributed to a cAMP protein kinase-mediated effect [64] which is consistent with the known increase of cAMP by DP receptor stimulation [39].

Surface expression of P-selectin is upregulated in activated platelets and plays an important role in platelet-leukocyte-endothelial interaction. Conformational change of GPIIb/IIIa to a high affinity state for fibrinogen enables thrombus formation. Activation of the DP receptor abrogated the agonist-induced P-selectin expression and GPIIb/IIIa activation. All effects elicited by activation of the DP receptor were counteracted by the DP receptor antagonist BWA868c and by laropiprant at 1 µmol/L. These findings translated to the inhibitory effects of DP receptor activation on thrombogenesis under flow condition. Laropirant at 1 µmol/L had no effect on its own on thrombus formation, while counteracting the inhibition induced by DP receptor stimulation, which suggests that laropiprant has potential pro-thrombotic effects *in vitro*. However, residual activity of laropiprant on pro-aggregatory TP receptors has been purported [25] which might compensate for the loss of anti-aggregatory DP activity. We have further advanced this notion by showing that platelet aggregation, Ca^2+^ mobilization, P-selectin expression and GPIIb/IIIa activation induced by the TP receptor agonist U46619 were completely blocked by laropiprant at a concentration of 10 µmol/L. Interestingly, laropiprant already at a concentration of 1 µmol/L markedly attenuated collagen-induced platelet aggregation (Figure 1 D). Lai et al [25], [65], described an inhibitory effect on collagen-induced platelet aggregation *ex vivo* when plasma levels of laropiprant were about 5 µmol/L. Collagen has been found to induce platelet aggregation partially through TXA_2_ formation leading to TP receptor activation [66], and we have observed that the TP antagonist SQ29548 (1 µmol/L) exhibited similar inhibitory effect on collagen-induced aggregation as laropiprant (n = 4, data not shown). These data suggest that laropiprant attenuates collagen-induced platelet aggregation by blocking TP receptors. In contrast, no effects on the IP or EP4 receptors were observed in assays of platelet aggregation and Ca^2+^ flux, showing that the promiscuity of laropiprant does not extend to these inhibitory platelet receptors.

An interesting novel finding of the current study is that laropiprant also attenuates EP3-mediated responses. PGE_2_ has a biphasic, concentration-dependent effect on platelet aggregation. While at high concentrations it counteracts aggregation and thrombus formation of human platelets via activation of the EP4 receptor [14]–[17], at lower concentrations it aggravates agonist-induced aggregation by activation of the EP3 receptor, by eliciting Ca^2+^ mobilization, decreased VASP phosphorylation and enhanced P-selectin expression [12]. An EP3 antagonist has been proposed to be useful for antithrombotic therapy [12]. We observed that laropiprant antagonized the EP3-mediated enhancement of ADP-induced platelet aggregation, P-selectin expression and GPIIb/IIIa activation. Interestingly, inhibition of prostaglandin synthesis using acetylsalicylic acid attenuated the sulprostone-induced facilitation of platelet aggregation and abolished the inhibitory effect of laropiprant. Sulprostone acts as an EP3/EP1 agonist, however, the EP1 antagonist SC51322 did not abrogate the stimulatory effect of sulprostone. In addition, we found that the TP antagonist SQ29548 reduced the effect of sulprostone and did not further enhance the effect of laropiprant. Therefore, in agreement with previous reports [12] we hypothesize that EP3 activation triggers TXA_2_ release which in turn activates TP receptors to promote platelet aggregation, and TP blockade by laropiprant disrupts this signaling cascade. Similar observations were made with regard to regulation of vascular tone by the EP3 receptor: vasoconstriction induced by EP3 receptor activation is mediated through increasing TP-mediated signaling in guinea-pig aorta [67], and a TP antagonist attenuates vasoconstrictor responses to EP3 agonist in rat mesenteric artery [68]. Therefore, laropiprant might be capable of opposing EP3/TP-mediated vasospasm in vascular inflammation in patients with atherosclerosis.

Together, our results suggest that laropiprant can interfere with platelet aggregation by inhibiting pro-aggregatory targets, TP and EP3 receptors. This was further substantiated by our results of *in vitro* thrombogenesis of whole blood under flow condition. At the concentration of 10 µmol/L, laropiprant inhibited thrombogenesis to an extent comparable to acetylsalicylic acid (1 mmol/L). Therefore, it can be assumed that, at this concentration, laropiprant acts as a TP antagonist and hence inhibits thrombogenesis. Even though the dose given in combination with niacin is 40 mg/day, which results in peak concentrations of 1.5–2.5 µmol/L [25], [69], laropiprant up to a dose of 600 mg per day is well tolerated in healthy subjects and a multiple oral dose of 200 mg/day for 10 days results in a peak plasma concentration of 11.4 µmol/L [25]. Our *in vitro* results demonstrate the potential of laropiprant to antagonize DP, TP and EP3 mediated effects; however, whether this is clinical relevant depends on the endogenous PGD_2_, thromboxane and EP3 agonist activity.

It has been shown that niacin and its metabolite niceritrol inhibit platelet aggregation *in vitro* [40], [41] and incubation of whole blood with niacin at a clinically relevant concentration increases the concentrations of PGD_2_, PGE_2_ and TXA_2_ [41]. Our results show that niacin causes a profound inhibition of thrombus formation *in vitro*; however, prostaglandins such as PGD_2_ do not seem to be involved in this process, since pre-treatment of blood with acetylsalicylic acid as well as laropiprant did not counteract this effect. Therefore, the mechanisms involved in niacin-induced inhibition of platelet aggregation remain to be investigated.

In conclusion our data show that laropiprant at a low concentration antagonizes the effects of DP receptor activation on platelets while it does not reverse the anti-aggregatory effect of niacin. However, at a concentration that has been shown to be well tolerated [25], laropiprant attenuates TP and EP3 receptor function and effectively inhibits thrombus formation. Although further *in vivo* studies with oral application of the drugs are needed, our *in vitro* findings suggest that higher concentrations of laropiprant may have beneficial effects with respect to platelet function and thrombotic events in vascular disease.
